# *Sclerospora graminicola* Suppresses Plant Defense Responses by Disrupting Chlorophyll Biosynthesis and Photosynthesis in Foxtail Millet

**DOI:** 10.3389/fpls.2022.928040

**Published:** 2022-07-12

**Authors:** Baojun Zhang, Xu Liu, Yurong Sun, Lin Xu, Zhixian Ren, Yaofei Zhao, Yuanhuai Han

**Affiliations:** ^1^College of Plant Protection, Shanxi Agricultural University, Taiyuan, China; ^2^College of Agriculture, Shanxi Agricultural University, Taiyuan, China; ^3^Shanxi Key Laboratory of Minor Crop Germplasm Innovation and Molecular Breeding, Taiyuan, China

**Keywords:** foxtail millet, *Sclerospora graminicola*, transcriptome, chloroplast, photosynthesis

## Abstract

Downy mildew of foxtail millet is an important oomycete disease caused by *Sclerospora graminicola*, affecting the yield and quality of the crop. Foxtail millet infected with *S. graminicola* exhibit symptoms of leaf yellowing and leaf cracking. To uncover the pathogenic mechanism of this disease, we explored the effects on chlorophyll synthesis and photosynthesis of foxtail millet leaves infected by *S. graminicola*. An elite foxtail millet variety, JG21, susceptible to *S. graminicola*, was used as for this study. *S. graminicola* inhibited chlorophyll synthesis and caused loose mesophyll cell arrangement. In addition, some cells were severely vacuolated in *S. graminicola*-infected foxtail millet leaves at the early stages of infection. *S. graminicola* could invade the mesophyll cells through haustoria which destroyed the chloroplast structure at the middle stages of infection causing significant accumulation of osmiophilic particles (OPs) and disintegrated chloroplast grana lamellae. Furthermore, foxtail millet leaves split longitudinally at the later stages of infection. Chlorophyll and carotenoid contents in infected leaves decreased significantly compared with those in the control. Net photosynthetic rate (Pn) of leaves and stomatal conductance showed a downward trend, and intercellular carbon dioxide concentrations increased significantly following the infection with *S. graminicola*. A total of 1,618 differentially expressed genes (DEGs) were detected between the control group and the treatment groups using RNA sequencing (RNA-Seq) among S1–S5 stages. DEGs associated with “photosynthesis” and “light reaction” were enriched. Gene expression patterns showed that 91.3% of 23 genes related to chlorophyll synthesis and photosynthesis, were significantly down-regulated than the control during S1–S5 stages. Based on the gene expression dataset, weighed gene co-expression network analysis (WGCNA) with 19 gene co-expression modules related to photosynthesis revealed six hub genes related to chlorophyll synthesis, which were suppressed during infection. The results suggest that infection of *S. graminicola* led to weak chlorophyll synthesis and rapid chloroplasts disappearance in foxtail millet. The defense responses and resistance of foxtail millet to *S. graminicola* were inhibited because chloroplast structure and function were destroyed in leaves, and the sexual reproduction in *S. graminicola* could be completed rapidly.

## Introduction

Chloroplasts are crucial players in plants and serves as factories of photosynthesis, *via* which light energy is converted into chemical energy. In addition, chloroplasts are the sites of biosynthesis of amino acids, fatty acids, secondary metabolites, and plant hormones (Poonam et al., 2015; Sun and Zerges, 2015). Chloroplasts also coordinate plant defense responses as environmental sensors and signaling hubs. Pathogen-associated molecular patterns (PAMPs) triggered immunity (PTI) immune responses are triggered by PAMPs following perception of pathogenic threats from the environment by plants. Chloroplast-derived reactive oxygen species (cROS) play a key role in the establishment of effective plant immunity. ROS bursts initiate a signaling cascade to the nucleus through retrograde signaling, which leads to the expression of defense-related genes due to Ca^2+^ draining from the lumen to the matrix through calcium sensing receptors (CASR) in the chloroplast thylakoid (Chan et al., 2016; Yang et al., 2021). Studies have shown that cROS production induced by PTI and effector-triggered immunity (ETI), superoxide anion radical (O_2_^–^) production by photosystem I (PSI), and singlet oxygen (_1_O_2_) production by photosystem II (PSII) could lead to lipid peroxidation of cell membrane, in turn inducing hypersensitive responses (HRs) (Zoeller et al., 2012; Dogra et al., 2019). Furthermore, chloroplasts are the main organelles of a plant cell that synthesize salicylic acid (SA) and jasmonic acid (JA), the primary defense hormones involved in both local and systemic resistance in plants (Stael et al., 2015). Recently, some plant proteins have been demonstrated to have dual protein localization in plasma membrane (PM) and chloroplast, which could also be re-localized from the PM to chloroplasts, and activate defense signaling pathways against plant pathogens. Information is transmitted to the nucleus from chloroplasts through retrograde signaling pathways upon pathogen attack (Medina-Puche et al., 2020).

Since chloroplasts play important roles in plant immunity, chloroplasts have become the target of pathogens (Littlejohn et al., 2020). Pathogens alter the expression of nucleus-encoded chloroplast genes (NECGs) leading to the inhibition of chloroplast development. Kupeevicz (1947) reported that viruses and plant pathogens could alter chlorophyll accumulation following infection (Kupeevicz, 1947). *Rhizoctonia solani* infection can deform host chloroplasts and weaken photosynthesis, in turn facilitating fungal infection and disease development (Ghosh et al., 2017). Furthermore, *Pseudomonas syringae* effectors could reprogram the differential expression of NECGs in *Arabidopsis thaliana* (Zabala et al., 2015). The pathogen effectors could alter thylakoid membrane structure in chloroplasts, and inhibit receptors in plant defense signaling, including SA, nitric oxide (NO), and reactive oxygen species (ROS). Cysteine protease HopN1 secreted by *Pseudomonas aeruginosa* is localized in chloroplasts and both degrades PsbQ and inhibits PSII activity in chloroplast preparations (Rodríguez-Herva et al., 2012). Bacterial effectors *Hopl1*, *HopK1*, and *AvrRps4* target the chloroplast and influence its function (Jelenska et al., 2007; Li et al., 2014). A haustorium-specific protein (Pst_12806) from the wheat stripe rust fungus interacts with the C-terminal Rieske domain of the wheat TaISP protein (a putative component of the cytochrome b6-f complex), which impairs photosynthesis and reduces ROS accumulation (Xu et al., 2019). A *Plasmopara viticola* RXLR31154 effector targets a chloroplast protein PsbP (Liu et al., 2021).

Recent reports have shown that viruses can modify the retrograde signaling pathway transducing signal from chloroplasts to nucleus (Caplan et al., 2015). The C_4_ protein encoded by tomato yellow leaf curl virus (TYLCV) with two subcellular targeting signals, an N-myristoylation site and a chloroplast transit peptide, could re-localize from the PM to chloroplasts and bind to calcium sensing receptor (CAS) in thylakoid transmembrane, interfering with the chloroplast retrograde pathway upon activation of plant defense, interfering with the chloroplast-dependency (Medina-Puche et al., 2020). Coat protein (CP) encoded by cucumber mosaic virus (CMV) mutants cause differential expression of chloroplast and photosynthesis related genes (CPRGs) in mosaic leaves of tobacco plants infected with CMV (Mochizuki et al., 2014a).

Chloroplasts are at the crossroads of photosynthesis, pathogen infection, and plant defense processes, and is the emerging battlefield in plant–microbe interactions (Lu and Yao, 2018). Downy mildew of foxtail millet [*Setaria italica* (L.) P. Beauv] is caused by *Sclerospora graminicola* (Sacc). The incidence of downy mildew disease in foxtail millet in China ranges between 20 and 30%; however, the infection rates in some susceptible varieties can exceed 70% in some years (Li et al., 2020). Symptoms of the disease include rotten buds at the germination stage, downy mildew on the abaxial leaf surface, known as “gray back,” and witches’ broom, also known as “hedgehog panicle,” Importantly, the infected foxtail millet showed etiolated leaves. Our study focused on the key factors related to chlorophyll synthesis and photosynthesis during interactions between foxtail millet and *S. graminicola*. The present study also explored the key factors and pathways related to sexual development in *S. graminicola*. The results of the present study could enhance our understanding of downy mildew disease of foxtail millet and its infection mechanisms, and facilitate the development of effective control strategies.

## Materials and Methods

### Foxtail Millet Varieties, Inoculation, and Planting

Foxtail millet downy mildew oospores were isolated from experimental field at Shanxi Agricultural University, China. Brown “infected leaf” materials in late autumn were selected and then were placed in the shade to dry, crushed, and sieved to obtain oospores, and subsequently stored at −20°C. The host plant material was from a *S. graminicola*-susceptible variety, Jingu21 (JG21), provided by the Germplasm Bank of the Institute of Bioengineering, Shanxi Agricultural University. JG21 seeds were sown with mixture of oospores and fine soil (1:500). And all materials were grown from May to October of 2019 on the field located in Taigu, Shanxi, China (37°25′ N, 112°35′ E). Monthly average temperature from May to October was 11.5–25.4°C and total rainfall was 301.3 mm. The field be irrigated before sowing and the seeds were planted at a depth of 1–2 cm, with rows spacing at 40 cm and plants 20 cm. Plots were arranged based on a random block design and the area of each block was 2 m × 2.5 m. The field experiment followed the standard local agronomic foxtail millet management practice. Each treatment group had three biological replicates, healthy and disease-free “JG21” individuals as the control.

### Microscopic Observation

Foxtail millet grown in field at the 13-leaf and the stamens and pistil differentiation stage, and the new leaves were listed and marked. In the subsequent sampling process, the marked leaves were drawn to ensure that the tested materials were at the same growth point at different stages of the plants. Samples were collected every 3 days and a total of five times were drawn. The corresponding stages were S1, S2, S3, S4, and S5, respectively. The control group was marked as CK1–CK5, and the treatment group was marked as TG1–TG5, respectively. Two samples of 1 cm^2^ were cut form infected leaves or the control, and one sample was fixed in FAA (70% alcohol: formaldehyde: glacial acetic acid = 16:1:1), dehydrated, transparent, impregnated, and embedded, and used for preparing paraffin sections as described (Yang, 2006). The sections were observed under a fluorescence microscope (BX53, OLYMPUS) after stained in 0.05% aniline blue staining solution for 20 min. The other sample was fixed with 2.5% glutaraldehyde, prepared according to the method of Yang et al. (2013), cut into 80–100 nm ultra-thin sections, and observed under a transmission electron microscope (TEM) (JEM-1400, JEOL, Tokyo, Japan).

### Determination of Chlorophyll Content and Physiological Biochemistry

The marked leaves were collected at the S1–S5 stages with the midrib tissue removed, and stored at −20°C for later use. The contents of chlorophyll, carotenoid, and soluble sugar, phenylalanine ammonia lyase (PAL) activity and NO content were determined using a plant chlorophyll determination kit (colorimetry, 50T/48S), a plant carotenoid test kit (colorimetry, 50T/48S), a soluble sugar content determination kit (colorimetry, 50T/48S), a PAL test kit (phenylalanine colorimetry, 50T/48S), and a plant NO test kit (nitrate reductase method, 50T/48S) according to the instructions, respectively, which were purchased from Nanjing Jiancheng Bioengineering Institute, Nanjing, China.

### Determination of Photosynthesis

Net Pn, transpiration rate (Tr), intercellular CO_2_ concentration (Ci), and stomatal conductance (Gs) in infected JG21 leaves were measured at the middle jointing, late jointing, booting, heading, and filling stages, numbered T1–T5, using a portable photosynthesis meter (LI6400XT, America LI-COR, Lincoln, NE, United States). The leaves were measured in a leaf chamber (6 cm^2^) under photosynthetically active radiation of 1,500 μmoL/(m^2^⋅s), an ambient CO_2_ concentration of 380–410 μmoL CO_2_/moL air between 09:00 and 11:30 am. Water use efficiency (WUE) was calculated based on the formula WUE = Pn/Tr.

### RNA Sequencing and Transcriptional Analysis

Infected JG21 leaves were collected at S1–S5, dipped immediately in liquid nitrogen, and then stored at −80°C. Leaves of healthy plants were collected at the similar developmental stages for use as controls. Three replicates were obtained at each stage, yielding five extractions with 30 samples in total. Total RNA was extracted from 500 mg infected and healthy leaves at five developmental stages using Trizol method (Rio et al., 2010). The RNA samples were separated on 1% (w/v) agarose gel, and RNA integrity and concentrations were checked using an Agilent 2100 Bioanalyzer (Agilent Technologies, Inc., Santa Clara, CA, United States).

A cDNA library was constructed using a NEBNext Ultra RNA Library Prep Kit for Illumina (NEB, E7530, Ipswich, United States) according to the manufacturer’s instructions, and sequenced on an Illumina Hiseq platform by Shanghai Majorbio Bio-Pharm Technology Co., Ltd, Shanghai, China. The generated sequencing data were evaluated using FastQC, and the adapter and low-quality reads (including more than 10% reads with N removal and more than 50% reads with Q ≤10 base number of the whole reads) removed. The filtered clean data were compared against the foxtail millet *xiaomi* genome^1^ using Hisat2. DESeq2 was used to assess differential expression between sample groups (Love et al., 2014). The DEGs were identified based on the following criteria: | log_2_FC (fold change) | ≥ 1 with *p*-value < 0.05. DEGs were functionally annotated using the Kyoto Encyclopedia of Genes and Genomes (KEGG) database^2^ and the Gene Ontology (GO) database.^3^ The Pheatmap package min R was used to generate expression Heatmaps.

### Analysis of Differentially Expressed Genes Related to Chlorophyll Synthesis and Photosynthesis

The genes encoding chlorophyll biosynthesis enzymes, core proteins of photosystems II, photosystems I, antenna protein and the cytochrome b6f complex were screened by enrichment theory of KEGG pathways. The heatmap was illustrated using R v3.4.4, and the levels of expression of associated genes in infected leaves at the S1–S5 stages were analyzed. TBtools (Toolbox for Biologists) v1.0971 (Chen et al., 2020) was used to analyze the *cis*-acting elements of chlorophyll synthesis-related genes in the transcriptome.

### Identification and Functional Analysis of Key Modules of Chlorophyll Synthesis

The weighted gene co-expression network and related modules were constructed using R v3.4.4 and the weighed gene co-expression network analysis (WGCNA) package in R v1.6.6 based on a gene expression matrix consisting of 30,487 genes from the transcriptomes of 30 samples. Soft thresholding was calculated using the pick Soft Threshold function in the WGCNA package, and the power value of the optimal network construction selected. A scale-free network was built based on block wise Modules, and the correlation between chlorophyll content and co-expression modules analyzed. Annotated networks were visualized in Cytoscape (Langfelder and Horvath, 2008). The hub genes that were related to chlorophyll synthesis were screened.

### Quantitative Real-Time PCR Analysis

The six hub genes were used for qRT-PCR verification. Extracted RNA was purified and reverse transcribed into cDNA using a PrimeScript™ RT reagent Kit with gDNA Eraser (Takara, Beijing, China). The primers used are listed in Supplementary Table 1. TB Green^®^ Premix Ex Taq^®^ II (Takara, Beijing, China) was used for the quantitative fluorescence assay. qRT-PCR amplifications were performed in 20 μL reaction volumes consisting of 10 μL of 2 × SG Fast qPCR Master Mix, 0.4 μL of each primer, 500 ng cDNA template, 2 μL DNA buffer, and PCR-grade water. The PCR setup was as follows: initial denaturation at 95°C for 3 min, followed by 40 cycles of denaturation at 95°C for 3 s, and annealing/extension at 60°C for 30 s. The actin gene was used as an internal control, and relative gene expression was calculated using the 2^–ΔΔCT^ method. The experiments were repeated at least three times.

## Results

### *Sclerospora graminicola* Infection of Foxtail Millet Leads to Leaf Malformation

**FIGURE 1:**
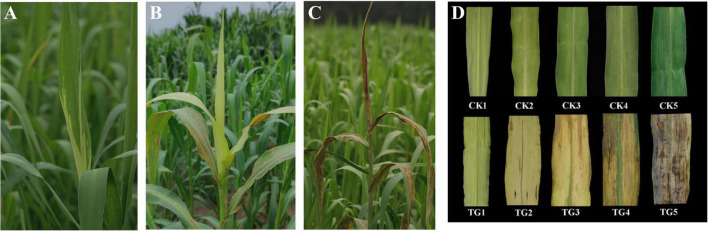
Symptoms of foxtail millet infected by *Sclerospora graminicola*. **(A)** The leaf of infected plants at S1 stage; **(B)** the leaf of infected plants at S3 stage; **(C)** the leaf of infected plants at S5 stage; **(D)** morphological characteristics of healthy leaves and infected leaves at S1–S5 stages.

At the booting stage, the *S. graminicola* infected plants showed curled up new leaves, which became tube-like and orientated upright, with the basal areas of leaf blade etiolated, affecting the growth and development of young panicles (Figure 1A). Gradually, all the new leaves were etiolated (Figure 1B). Then was the critical period of oospore formation for the pathogen *S. graminicola*. With the development of oospores, the host leaves turn brown and split longitudinally, and a large number of oospores were scattered into the field (Figure 1C). We observed the growth of the top second leaves, and the development of foxtail millet. The new leaves were initially light green and had low chlorophyll content. With chlorophyll synthesis and chloroplast development, the structure and function of chloroplasts improved gradually and the leaves developed into functional dark green leaves (Figure 1D). The top second infected leaves were similar to the healthy leaves in the initial stage of leaves development; however, the infected leaves became deformed, thick, and wide, and then yellowed and browned in the later stages of infection. Proliferation and sexual reproduction of *S. graminicola* led to the inhibition of chloroplast development.

### *Sclerospora graminicola* Infection of Foxtail Millet Affected Chloroplast Development

**FIGURE 2:**
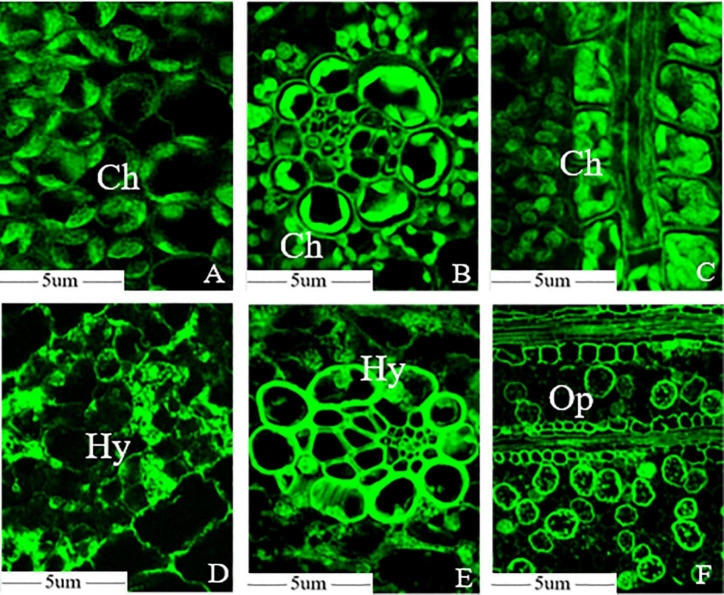
*Sclerospora graminicola* destroyed the mesophyll cells of foxtail millet. **(A)** Mesophyll cell microstructure of foxtail millet leaves; **(B)** bundle sheath cells microstructure of foxtail millet leaves; **(C)** transverse sections microstructure of foxtail millet leave; **(D)** mesophyll cell microstructure of infected leaves; **(E)** bundle sheath cells microstructure of infected leaves; **(F)** transverse sections microstructure of infected leaves. Ch, chloroplast; Hy, mycelium; Op, oospore.

Optical microscopy observation revealed that mesophyll cell structures remained intact and the chloroplasts were scattered in the cytoplasm with oval or sub-globose shapes, and some were close to the inside of the cell wall in the healthy leaf (Figure 2A). The Kranz structure were obvious in the leaves of foxtail millet, and the number of chloroplasts in vascular bundle sheath cells were higher than that in mesophyll cells (Figure 2B). Transverse sections of leaves revealed that the vascular bundle sheath cells surrounded the vascular tissue and the mesophyll cells were arranged regularly (Figure 2C). However, chloroplast development was significantly inhibited in infected leaves at the initial stage, with the *S. graminicola* hyphae expanded between the mesophyll cells and fully developed chloroplasts hardly observed in the mesophyll cells (Figure 2D). The expansion of the hyphae destroyed the mesophyll cell walls, leading to the intracellular structural disorder and extravasation of cellular contents. Chloroplast development in vascular bundle sheath cells was also inhibited significantly; however, the cell walls of vascular bundle sheaths had no apparent phenotypic alterations (Figure 2E). The mesophyll cell wall was completely destroyed, with only vascular bundle tissue remained and a large number of oospores formed between leaf veins at the late stages of disease development (Figure 2F).

**FIGURE 3:**
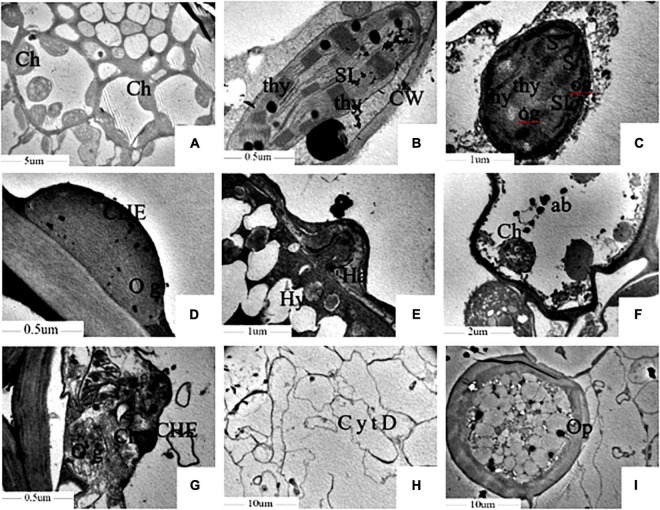
*Sclerospora graminicola* inhibited the development of chloroplast of foxtail millet. **(A,B)** Are uninfected leaves; **(C–I)** are infected leaves. Ch, chloroplast; thy, thylakoid; SL, stromal lamella; Hy, mycelium; Ha, haustorium; S, starch granule; og, osmiophilic granule; ab, apoptotic corpuscle; Op, oospore; CW, cell wall; CHE, chloroplast membrane; CytD, cytoplasmic degradation.

The microstructure showed that the Kranz anatomy constituted vascular bundles, the parenchyma sheath, and the surrounding mesophyll cells, which were clearly visible in healthy leaves under a TEM. The chloroplast structure was well developed. The thylakoids in the chloroplast, which had high proportions of stromal lamellae and granal lamellae, were arranged in an orderly manner, and the inner and outer membranes of chloroplasts were intact (Figures 3A,B). In contrast, in infected foxtail millet leaves, the mesophyll cell wall was distorted, the intercellular space volume increased, and the PM was invaginated under the action of haustoria of *S. graminicola* (Figure 3C). Chloroplasts were small and deformed in foxtail millet leaves infected with *S. graminicola*, and the inner and outer membranes of chloroplasts were unclear, the grana were loosely and irregularly arranged, the lamellar structure was disordered, a large number of osmiophilic granules were formed, and starch grains were accumulated in the chloroplast (Figures 3D,E). In addition, other organelles were destroyed, with a large number of apoptotic bodies formed, along with the expansion of hyphae of *S. graminicola* (Figure 3F). The chloroplast membrane disappeared and the chloroplast was decomposed as the outer membranes of the chloroplasts ruptured (Figure 3G). The cell wall of mesophyll cells had become thin and broken, the cytoplasm degraded, and vacuoles formed in the late stage of infection of foxtail millet leaves with the formation of a large number of oospores (Figure 3H). The diameter of mature oospores was 40–50 μm. The thicker outer wall of oospores was the most obvious feature, with a diameter of 6–9 μm outer cell wall (Figure 3I).

### Decrease in Chloroplast Content in Foxtail Millet Leaves Infected With *Sclerospora graminicola*

**FIGURE 4:**
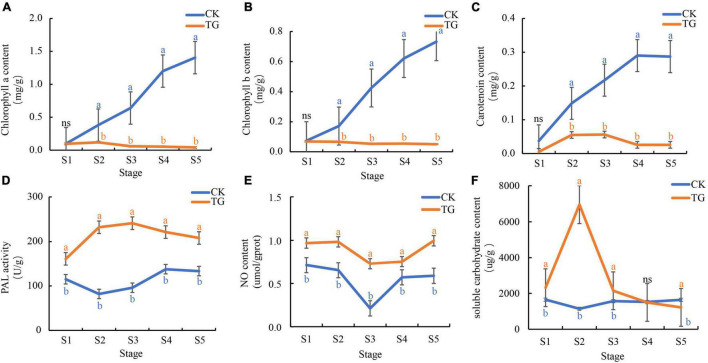
*Sclerospora graminicola* inhibited the substance accumulation or enzyme activity related to photosynthesis. **(A)** chlorophyll a content in CK and TG; **(B)** Chlorophyll b content in CK and TG; **(C)** carotenoid content in CK and TG; **(D)** PAL activity in CK and TG; **(E)** NO content in CK and TG; **(F)** soluble carbohydrate content in CK and TG. The letters indicate correlation significance at 5% probability (*P* < 0.05), the same below.

Chlorophyll a and b (Chl a/b) contents of leaves in the control increased gradually with chloroplast development and foxtail millet leaf development. Chl a and Chl b contents were 0.1–1.40 and 0.07–0.73 mg/g, respectively, and reached 1.40 and 0.73 mg/g, respectively, at the S5 stage (Figures 4A,B), whereas they were only 0.04–0.12 and 0.05–0.07 mg/g, respectively, in the leaves infected with *S. graminicola*. There was no significant difference with Chl a/b contents between the control and the treatment at the S1 stage. In addition, Chl a and Chl b contents did not change significantly over the S2–S5 stages in the treatment leaves, and the lowest Chl a and Chl b contents at the S5 stage were 0.04 and 0.05 mg/g, respectively, which were significantly different from those in the control. The carotenoid content trends in healthy leaves were similar to those of chlorophyll. The highest carotenoid content was 0.29 mg/g, at the S4 stage (Figure 4C), In contrast, carotenoid contents decreased at the S3 stage, and was only 0.03 mg/g at the S4 stage, in the infected leaves.

Phenylalanine ammonia lyase enzyme activity enzyme in foxtail millet leaves infected with *S. graminicola* was significantly higher than that in healthy leaves at the S1–S5 stages, and was the highest at the S3 stage, at 241.36 U/g (Figure 4D). NO content in foxtail millet leaves infected with *S. graminicola* was significantly higher than that in the control, with contents varying from 0.73 to 1.00 μmoL/gprot at S1–S5 (Figure 4E). Soluble sugar contents in the treatment groups increased at first and then decreased, and was the highest at the S2 stage, at 6953.27 μg/g (Figure 4F).

### Photosynthesis Was Inhibited in Foxtail Millet Leaves Infected by *Sclerospora graminicola*

**FIGURE 5:**
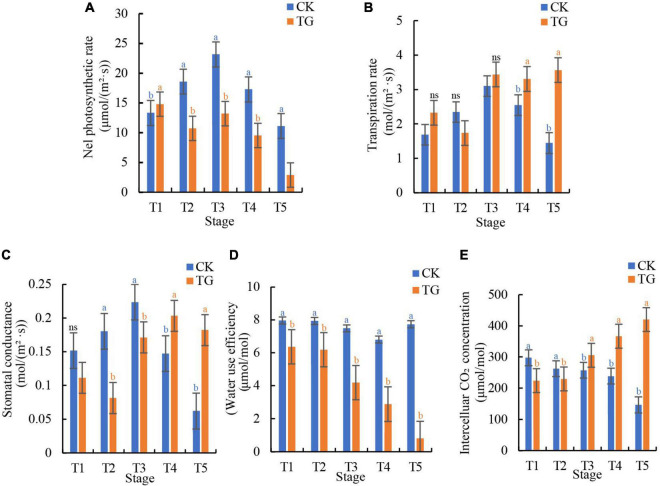
*Sclerospora graminicola* caused damage on the photosynthesis of foxtail millet. **(A)** The net photosynthetic rate of CK and TG at T1–T5 stages; **(B)** the transpiration rate of CK and TG at T1–T5 stages; **(C)** the stomatal conductance of CK and TG at T1–T5 stages; **(D)** the water use efficiency of CK and TG group at T1–T5 stages; **(E)** the intercellular CO_2_ concentration of CK and TG at T1–T5 stages.

As expected, photosynthesis in the infected plants was significantly affected. Pn values were 2.88–14.79 and 11.12–23.16 μmoL⋅m^–2^⋅s^–1^ in the TG and CK groups, respectively, which was a significant difference at 0.05 level (Figure 5A). Pn decreased significantly compared with CK at the T5 stage (2.88 μmoL⋅m^–2^⋅s^–1^), with the proliferation of hyphae in treatment groups. The highest Tr and Gs in foxtail millet leaves at the five stages were 3.56 and 0.22 moL⋅m^–2^⋅s^–1^, respectively, which were significantly different from those in the control at the same stage (Figures 5B,C).

Mesophyll cell structure in foxtail millet leaves infected with *S. graminicola* were destroyed, leading to significant increases in in Tr and Gs in foxtail millet leaves, and a decrease in Pn. at the T1–T5 stages, the WUE of foxtail millet leaves in the control group was 6.79–7.95 μmoL/moL, which was rather stable, whereas WUE in foxtail millet leaves decreased significantly from T1 to T5 stage, with the lowest value (0.81 μmoL/moL) observed at the T5 stage in the TG group (Figure 5D). Ci in infected leaves, which were 224.41–420.61 μmoL/moL at the five stages, increased in comparison with that in the non-infected controls. In addition, Ci at the T5 stage (420.61 μmoL/moL) was 186.7% higher than that in the control (146.70 μmoL/moL) (Figure 5E). The results showed that photosynthesis in foxtail millet leaves infected with *S. graminicola* was decreased compared with CK, whereas Gs and Ci increased with a decline in WUE.

### Differential Gene Expression in Foxtail Millet Induced by *Sclerospora graminicola*

In total, 1,012 GB clean data were obtained from 30 samples, which were filtered using Illumina 2 × 150 bp double-end sequencing, and the average Q30 was above 94.48%. A total of 1,553,993 transcripts were generated between 30 samples and *xiaomi* reference genome based on Trinity assembly (Supplementary Table 2).

**FIGURE 6:**
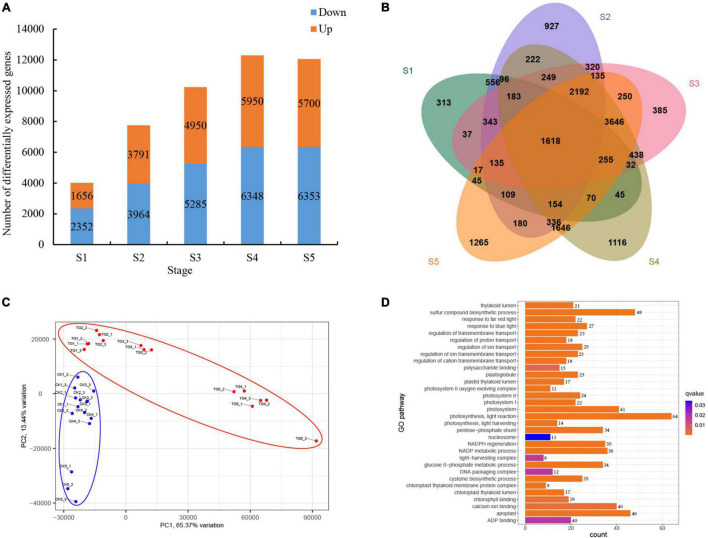
Analysis of differential gene expression in foxtail millet at different stages. **(A)** DEGs at different stages; **(B)** venn analysis of DEGs at different stages; **(C)** PCA analysis of samples; **(D)** GO enrichment analysis of common DEGs.

A total of 46,349 significantly DEGs were screened from healthy and *S. graminicola*-infected foxtail millet leaves in five stages (FDR < 0.001, | log_2_ fold change| ≥ 1). Among them, the largest number of DEGs was at the fourth stage, with 12,298 DEGs, including 5,950 up-regulated and 6,348 down-regulated genes. The numbers of DEGs in S3 and S5 were 10,235 and 12,053, respectively, and the numbers of DEGs in S1 and S2 were 4,008 and 7,755, respectively (Figure 6A). A total of 1,618 genes were differentially expressed at the five stages (Figure 6B). The transcriptional group data showed high similarity among the three biological replicates in each treatment, and significant differences between the control and treatment groups at the five stages. The contribution rates of PC1 and PC2 were 65.37 and 13.44%, respectively, based on principal component analysis (PCA) results (Figure 6C). Through GO enrichment analysis, the DEGs were more enriched in photosynthesis-related pathways, such as photosynthesis, light reaction (GO:0019684), response to blue light (GO:0009637), response to far red light (GO:0010218), photosynthesis, light harvesting (GO:0009765), chlorophyll binding (GO:0016168), light-harvesting complex (GO:0030076), photosystem (GO:0009521), photosystem I (GO:0009522), photosystem II (GO:0009523), chloroplast thylakoid lumen (GO:0009543), photosystem II oxygen evolving complex (GO:0009654), and chloroplast thylakoid membrane protein complex (GO:0098807). Among them, GO:0019684 enriched the largest number of genes, with a total of 64 genes (Figure 6D). The results revealed a total of 24 transcription factors (TFs) families were enriched in 32 GO terms with 94 genes encoding TFs in the 1,618 common DEGs) WRKY, MYB, HSF, ERF, and bZIP TFs play critical roles in plant disease resistance, and the expression analysis showed the genes expression levels were dramatically reduced in infected leaves during the S1–S5 stages. The results showed that the infection of *S. graminicola* could destroy the defense system of foxtail millet, and infringe the chlorophyll synthesis and photosynthesis of infected leaves, resulting in leaf yellowing and leaf longitudinal cracking (Supplementary Figure 1). In addition, Photosynthesis antenna proteins (ko00195), Photosynthesis (ko01200), Carbon metabolism (ko00561), beta-Alanine metabolism (ko00410), Glycolysis/Gluconeogenesis (ko00010), Pyruvate metabolism (ko00620), Glutathione metabolism (ko00480), Propanoate metabolism (ko00640), and Citrate cycle (TCA cycle) (ko00020) were enriched significantly based on the results of KEGG enrichment analysis for the common DEGs (Supplementary Figure 2).

### Expression of Chlorophyll Biosynthesis and Photosynthesis Related Genes in Response to Infection by *Sclerospora graminicola*

**FIGURE 7:**
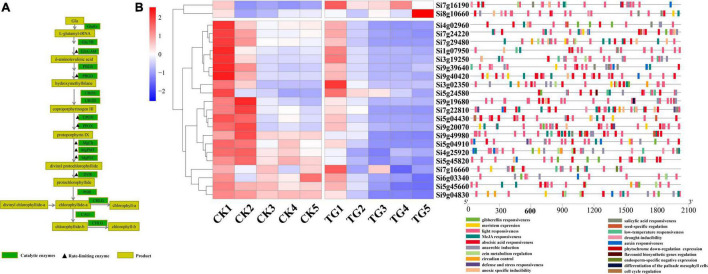
Analysis of expression of genes related to chlorophyll biosynthesis. **(A)** Chlorophyll synthesis pathway and related enzymes; **(B)** heat map and *cis* acting element analysis of genes related to chlorophyll synthesis at each stage.

Chlorophyll biosynthesis involves more than 15 enzyme-catalyzed reactions from L-glutamyl-tRNA to Chl a/b (Figure 7A). A total of 23 genes encoding chlorophyll synthesis-related synthase were obtained from the *xiaomi* genome, and their expression levels were analyzed at the five stages. According to the results, two cluster expression patterns were observed, and the expression levels of the 21 genes of the TG group were significantly lower than those in the control in Cluster2 in the S1–S5 stages, and only Si7g16190 and Si8g106602 genes were in Cluster1, which demonstrated an inverse trend.

Chlorophyll biosynthesis-associated genes showed higher levels of expression at the S1 and S2 stages than in the other stages in the control treatment, and tended to be stable at S3–S5. The results showed that chlorophyll synthesis and chloroplast rapid development in leaves through up-regulation of the expression levels of most of chlorophyll biosynthetic pathway genes at the early stages of leaf development. Furthermore, the overall gene expression levels were stabilized in functional leaves after the S3 stage. The expression of 21 genes were between 0.91 and 729.8 in foxtail millet leaves infected with *S. graminicola* in the S1 stage, and genes encoding chlorophyll biosynthesis enzymes were down-regulated significantly, at the S2–S5 stages, which disrupted chloroplast development. Analysis of *cis*-acting elements showed that all 23 genes had light responsive elements, and Si9g40420 and Si9g49980 had the highest proportions. In addition, hormone response elements, such as abscisic acid, gibberellin, auxin, methyl jasmonate, and SA and abiotic stress response elements were detected (Figure 7B).

**FIGURE 8:**
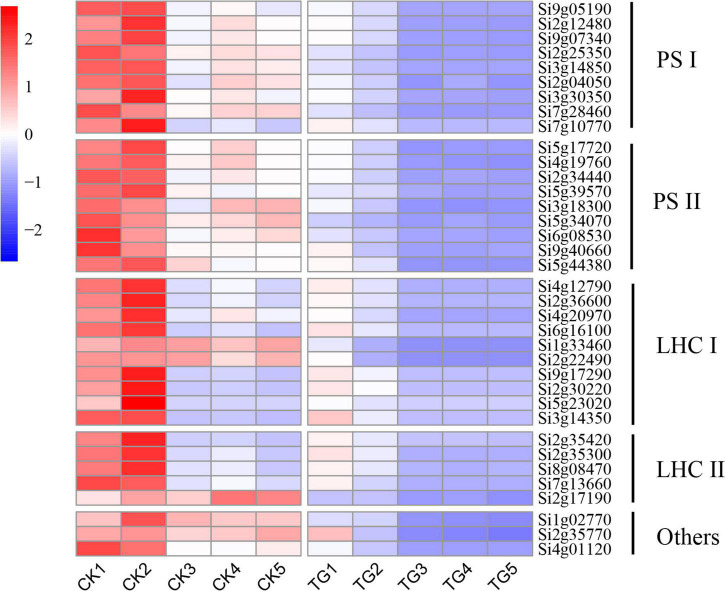
The expression heat map of photosynthesis-related genes at different stages.

According to the GO pathway enrichment analysis results, a large number of photosynthesis-related genes were enriched significantly (Figure 6D). The genes encoding photosystem II proteins (PsbO, PsbP, PsbQ1, PsbS, PsbW, or PsbY), photosystem I (PsaD, PsaE, PsaF, PsaG, PsaH, PsaK, PsaL, PsaN, or PsaO), antenna proteins (Lhca1, Lhca2, Lhca3, Lhca3, Lhca6, Lhcb3, Lhcb4.1, Lhcb5, or Lhcb7), and cytochrome b6f complex proteins (PetC or Lhcb7) were identified from the genome, and the associated gene expression patterns in foxtail millet leaves analyzed under *S. graminicola* stress. The results showed that the gene expression levels were lower in the TG group than in the control group in S1–S5 (Figure 8).

### Weighed Gene Co-expression Network Analysis Identified Hub Genes Responded to the Infection

**FIGURE 9:**
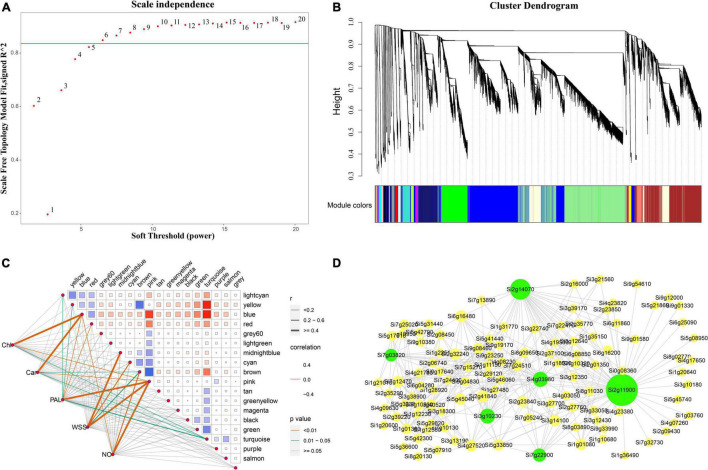
Co-expression networks of chlorophyll related genes based on Weighted Gene Co-expression Network Analysis (WGCNA). **(A)** Scale-free network model index under different soft thresholds, the abscissa represents the soft threshold, and the red line represents the optimal soft threshold; **(B)** gene clustering tree based on the topological dissimilarity matrix, and different colors represent different modules; **(C)** heat map of correlation between modules and traits, the abscissa and ordinate represent different modules, and the left part represents the sample traits; **(D)** correlation between modules and traits was analyzed and gene co-expression networks in the chlorophyll content-related gene module. Green in the Figure represents the core gene.

Weighed gene co-expression network analysis was applied to the transcriptomic data to explore the relationship between chlorophyll, carotenoid, and soluble sugar contents, NO, and PAL enzyme activity in foxtail millet and gene expression. The soft threshold β = 6 was determined by calculation (Figure 9A) and 30,487 genes were used to construct a co-expression network with 19 co-expression modules, among which the blue modules were highly related to chlorophyll synthesis with 7,215 genes (Figure 9B), with a correlation value of 0.86, and the highest negative correlations were observed in the pink and turquoise modules, with correlation values of −0.51 and −0.45, respectively (Figure 9C). Hotspot expression in gene regulatory networks was visualized using Cytoscape2.0 with weight >0.50 (Figure 9D). The six network hub genes were identified as key genes, and were annotated as chloroplast RNA binding protein, tRNA-dihydrouridine synthase, and others, in reference to rice and *Arabidopsis* databases (Table 1).

**TABLE 1:** Functional annotation of modular hub genes.

Candidate gene	Homologous genes in *O. sativa*	Gene function	Homologous genes in *A. thaliana*	Gene function
Si2g11900	XP_015646954	D-ribulose kinase	AT2G21370	D-ribulose kinase; Inactive Xylulose kinase 1
Si2g14070	XP_015611767	Flagellar radial spoke protein 5	AT2G27680	NAD (P)-linked oxidoreductase superfamily protein
Si4g03980	XP_015643470	Uncharacterized protein LOC4340112	AT5G27560	DUF1995 domain protein
Si3g10230	XP_015618439	Uncharacterized protein LOC4351751	AT4G40045	Transmembrane protein
Si7g03820	XP_015636752	Uncharacterized protein LOC4335364 isoform X1	AT3G63510	tRNA-dihydrouridine synthase
Si7g22900	XP_015637097	31 kDa ribonucleoprotein, chloroplastic	AT2G35410	Putative chloroplast RNA binding protein

### Verification Using Quantitative Real-Time PCR

**FIGURE 10:**
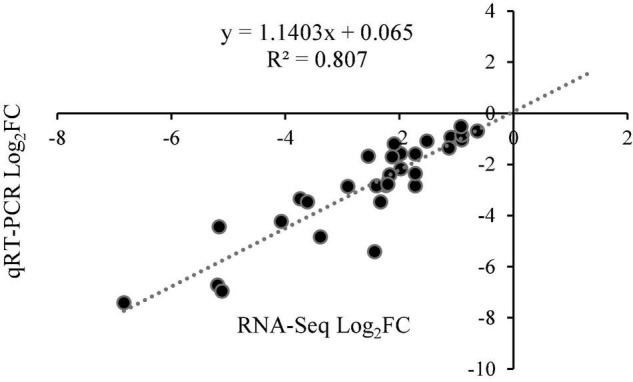
The correlation point map between RNA-Seq and qRT-PCR expression patterns.

To verify the reliability of the transcriptome data, six hub genes were selected for qRT-PCR verification. A comparison of the transformed transcriptome sequencing data and qRT-PCR data for the genes indicated that according to the expression trends, the RNA-Seq data and the qRT-PCR expression patterns were highly correlated, with a Pearson correlation coefficient (*R*^2^) of 0.807 (Figure 10 and Supplementary Figure 3), demonstrating the reliability of the RNA-seq results.

## Discussion

Chloroplasts have a crucial role in plant immunity against pathogens. Increasing evidence suggests that phytopathogens target chloroplast homeostasis as a pathogenicity mechanism (Ishiga et al., 2017). The chlorophyll contents of wheat infected by powdery mildew decreases significantly, with a highly significant correlation between chlorophyll content and disease index (Cao et al., 2009). In addition, in a previous study, disease severity was inversely correlated with chlorophyll content in the potato leaves (Gamliel et al., 1997). CPRGs were down-regulated in chlorotic tissues of tobacco infected with CMV (Mochizuki et al., 2014a), and *P. syringae* delivers effectors into the host cell *via* the type III secretion system (T3SS) and disrupts PSII by reprogramming NECGs (Zabala et al., 2015). The pathogen effector *AVRvnt1* binds a full-length chloroplast-targeted glycerate kinase (GLYK) isoform leading to lower GLYK accumulation in chloroplasts, and counteracting GLYK contribution to basal immunity (Gao et al., 2020). Kupeevicz first reported that viruses and other plant pathogens alter chlorophyll accumulation during infection (Kupeevicz, 1947). Leaf infected by *Fusarium oxysporum* f.sp. cubense in banana showing serious rupture of the PM and distortion of the chloroplasts and no starch granules are discernible, and many osmiophilic granules are present in chloroplasts (Dong et al., 2014).

In the present study, photosynthetic pigment content decreased significantly and chloroplast development was retarded in foxtail millet leaves infected with *S. graminicola*. Kupeevicz first reported that viruses and other plant pathogens alter chlorophyll accumulation during infection (Kupeevicz, 1947). The infected leaves with *F. oxysporum* f.sp. *cubense* in banana showed serious rupture of the PM and distortion of the chloroplasts and no starch granules are discernible, and many osmiophilic granules are present in chloroplasts (Dong et al., 2014). observation showed that the number of chloroplasts in the early stage of infected leaves was small, and some mesophyll cells and sheath cells were destroyed. Electron microscopic observations showed that chloroplast structure was markedly destroyed at the middle stage of infected leaves. And the transcriptome data also indicated that some photosynthesis genes and pathways were differentially altered by *S. graminicola*. Photosynthesis and chlorophyll biosynthesis-related genes expression levels are downregulated in infected leaves. Such as *Si5g04910* which encode Mg-protoporphyrin IX monomethyl ester cyclase in the chlorophyll synthesis pathway showed 67-fold reduction in TG compared with controls at S4 stage. *Si2g30220* which encode the light-harvesting complex I (LHC I) in photosynthesis showed 59.7-fold reduction in TG compared with controls at S4 stage. Arabidopsis infected by PstDC3000, over 1000 largely photosynthesis-related NECGs are down-regulated (Zabala et al., 2015). Tobacco leaves infected by CMV caused the expression of CPRGs reduced (Mochizuki et al., 2014b); A haustorium-specific protein (Pst_12806) from the wheat stripe rust fungus inhibits chloroplast-mediated accumulation of ROS and expression of resistance-related genes by interfering with the function of cytochrome b6f protein subunit TaISP in photosynthetic system, thus promoting colonization of stripe rust (Xu et al., 2019). These results suggest that pathogens could promote their own infection and extension plant growth through inhibited chlorophyll synthesis and the photosystem activity. Large amounts of mycelium and sporangium could reproduce in the leaves at the later stages of infection through microscopic observations.

A small number of chloroplasts retained in foxtail millet leaves would also be destroyed and degraded with proliferation of hyphae, which could indicate that chloroplasts might be attacked by the effector proteins secreted by *S. graminicola*. In our previous work, several effector proteins with predicted chloroplast localization were screened that potentially inhibit chlorophyll biosynthesis in young leaves (unpublished data). Researchers also elucidated the mechanism of C_4_ protein from the geminivirus *TYLCV* (Medina-Puche et al., 2020). C_4_ protein has two localization signals, an N-myristoylation site for PM localization, and an N-terminal transit peptide for import into chloroplasts (Rosas-Diaz et al., 2018; Lee et al., 2020), which transfers its localization from the PM to the chloroplast and binding to a thylakoid transmembrane protein, CAS, suppressed plant immune response. *RXLR31154* effector from *P. viticola* could target and stabilize a host protein, *PsbP*, leading to reduced defense response *in planta* and enhanced colonization (Liu et al., 2021).

Downy mildew of foxtail millet is an important class of systemic disease caused by *S. graminicola*. The oospores of *S. graminicola* could survive in dormant state in the soil for many years, which makes it a challenge to control. In a previous work, the biomass of oospores was up to 1.63 × 10^8^ copies/g in foxtail millet leaves infected with *S. graminicola*. Through quantitative RT-PCR at the “hedgehog like panicle” stage with decomposition of leaf mesophyll cells with only veins left. And then large numbers of oospores into soil that constitute potential source of initial infection in the subsequent year. The emergence of the “leaf etiolation” symptom represents a critical period in the development of *S. graminicola* oospores, which require high nutrient amounts, which inhibits chlorophyll biosynthesis during the formation of young leaves of foxtail millet. However, several plant-pathogen interactions explain the typical green islands observed on the infected plant tissues. It is likely that the flow of carbohydrates is forcibly directed into infected plant cells from neighboring uninfected plant cells (Bonfig et al., 2006; Xu et al., 2019). Therefore, *S. graminicola* primarily obtains nutrients from adjacent uninfected cells for its growth and development. In addition, autophagic cell death possibly occurs in *S. graminicola*, which accumulates and provides nutrient to satisfy various organism requirements, including oospore development, which has been observed in pathogen infection processes, for example, in *F. oxysporum* and powdery mildew (Wang et al., 2011; Khalid et al., 2019).

In conclusion, in the present study, we report the potential mechanisms involved and the influence of hub genes on the emergence of “leaf etiolation” in foxtail millet infected by *S. graminicola*. The mechanisms include the inhibition of the early stages of chlorophyll biosynthesis, destruction of chloroplast structure, reduction of rate of photosynthesis, inhibition of plant defense responses, and promotion of the sexual reproduction of oomycete pathogens. The results of the present study could enhance our understanding of the pathogenesis of *S. graminicola* and facilitate the formulation of strategies of controlling downy mildew disease in foxtail millet, primarily by interfering with the *S. graminicola* infection process and reducing oospore production.

## Data Availability Statement

The datasets presented in this study can be found in online repositories. The names of the repository/repositories and accession number(s) can be found below: https://www.ncbi.nlm.nih.gov; PRJNA852287.

## Author Contributions

BZ, YZ, and YH conceived of the study and designed the study. YS and LX performed the experiments and analyzed the data. ZR and XL interpreted the results. BZ and XL wrote the manuscript. All authors discussed the results and revised the manuscript.

## Conflict of Interest

The authors declare that the research was conducted in the absence of any commercial or financial relationships that could be construed as a potential conflict of interest.

## Publisher’s Note

All claims expressed in this article are solely those of the authors and do not necessarily represent those of their affiliated organizations, or those of the publisher, the editors and the reviewers. Any product that may be evaluated in this article, or claim that may be made by its manufacturer, is not guaranteed or endorsed by the publisher.

## Footnotes

1
http://foxtail-millet.biocloud.net/home

2
https://www.kegg.jp/

3
http://geneontology.org
