# Supplementary material for: Sclerospora graminicola Suppresses Plant Defense Responses by Disrupting Chlorophyll Biosynthesis and Photosynthesis in Foxtail Millet
Source: Front Plant Sci. 2022 Jul 12;13:928040. doi: 10.3389/fpls.2022.928040 (PMC9317951; doi:10.3389/fpls.2022.928040)
Supplement: Supplementary file 1 [file Image_1.pdf]

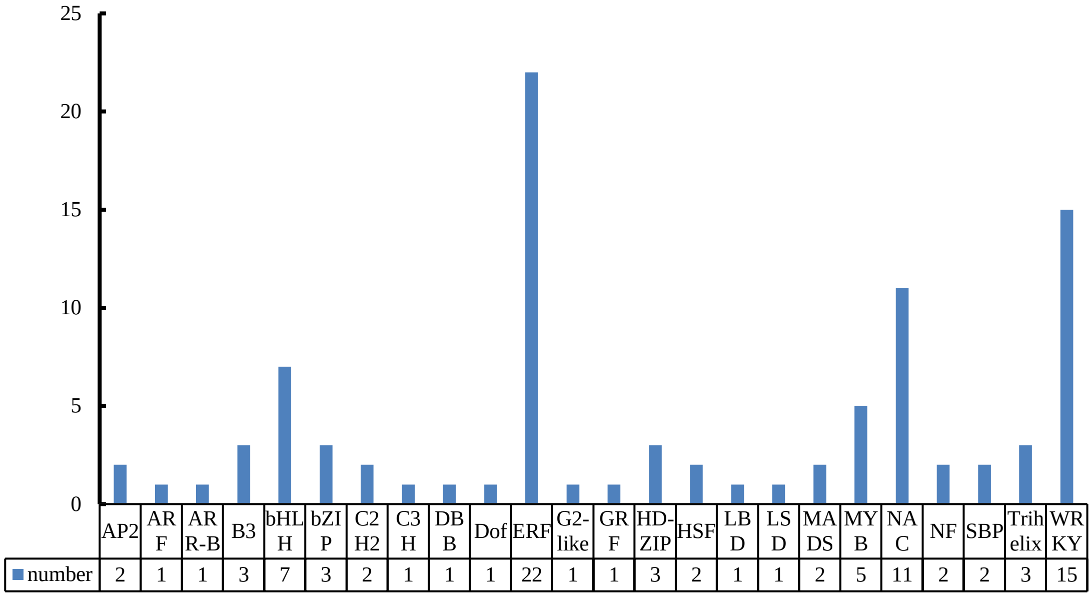

A

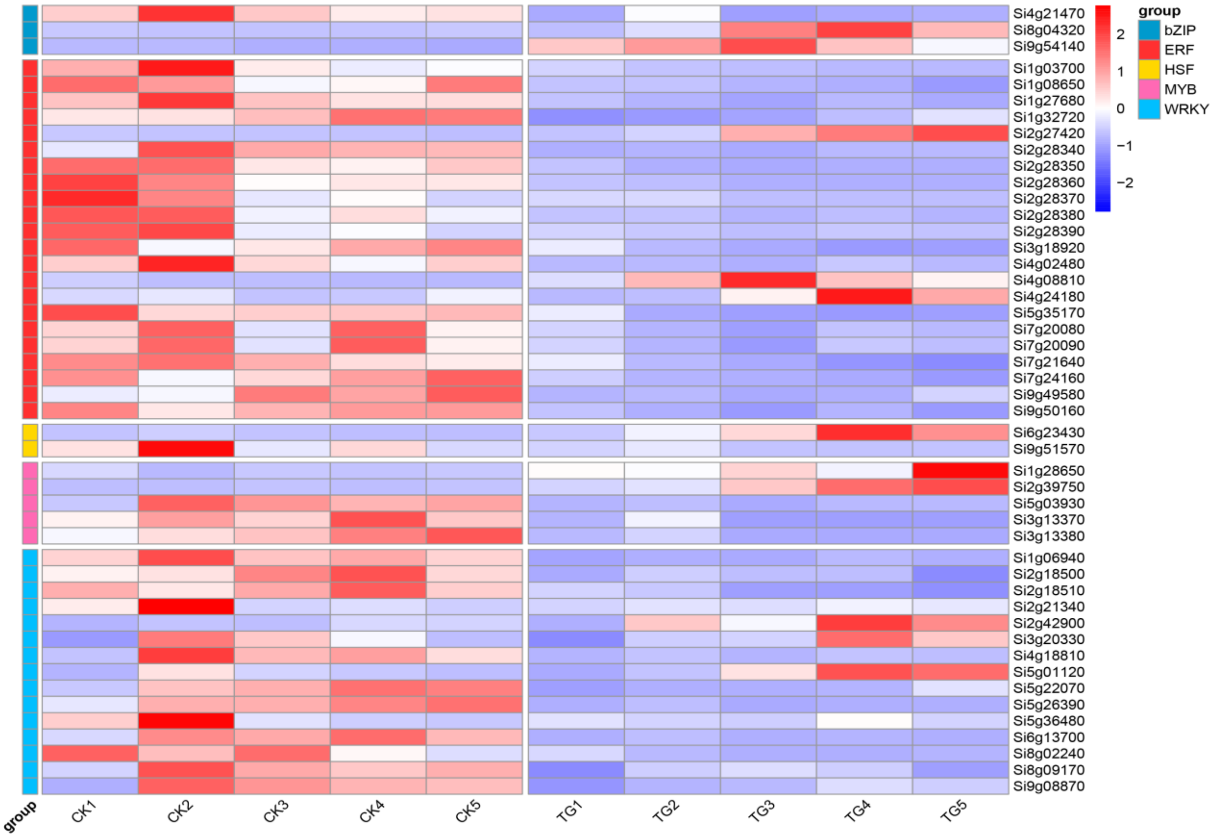

B

Supplementary Figure S1 Analysis of differential gene expressions of transcription factors (TFs) at S1-S5 stages

Note: A, the number of different TF families by GO enrichment analysis; B, The expression heat map of TF gene.
