# Supplementary figures and images for: Sclerospora graminicola Suppresses Plant Defense Responses by Disrupting Chlorophyll Biosynthesis and Photosynthesis in Foxtail Millet
Source: Front Plant Sci. 2022 Jul 12;13:928040. doi: 10.3389/fpls.2022.928040 (PMC9317951; doi:10.3389/fpls.2022.928040)

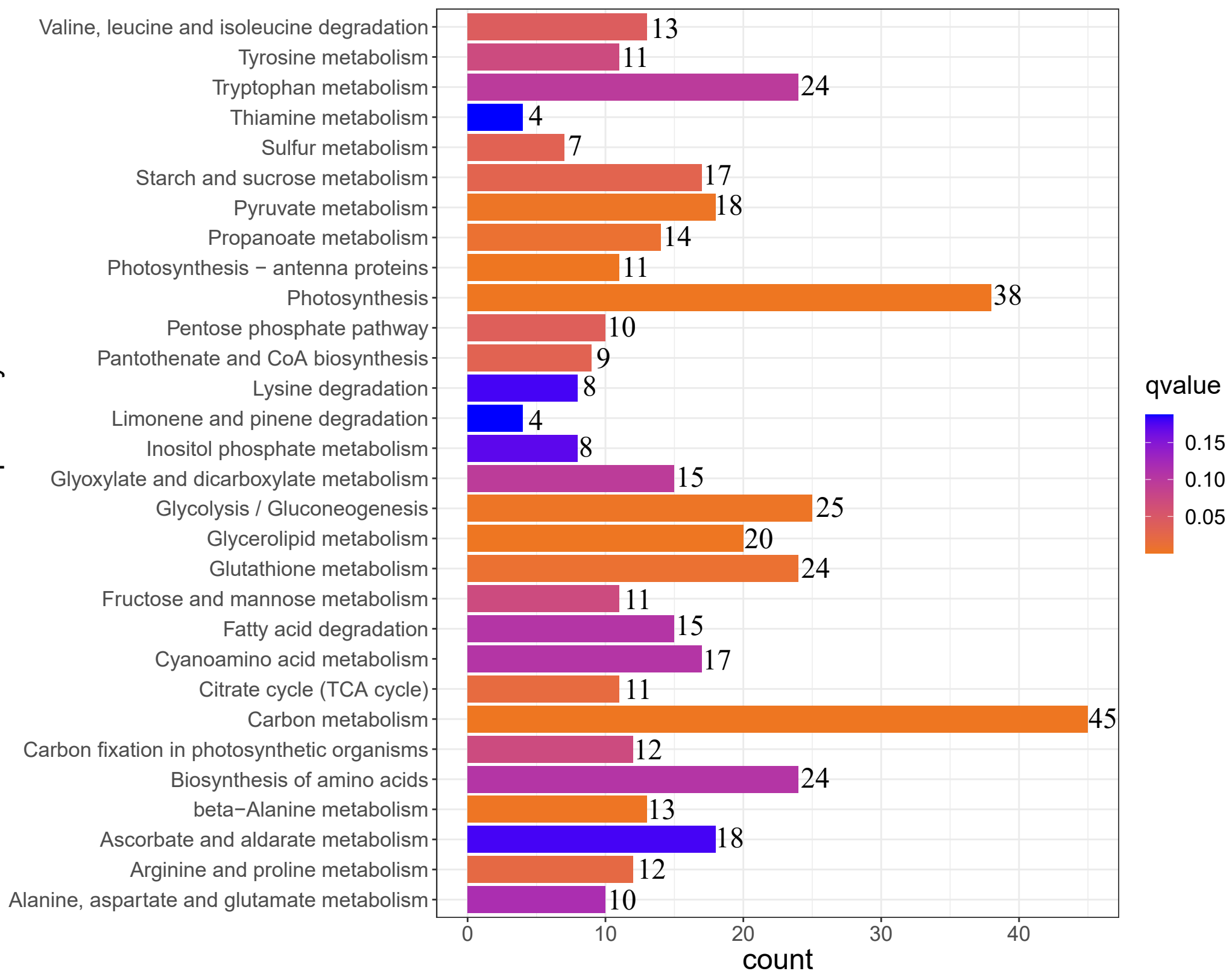

Supplement: Supplementary file 2 [file Image_2.pdf]

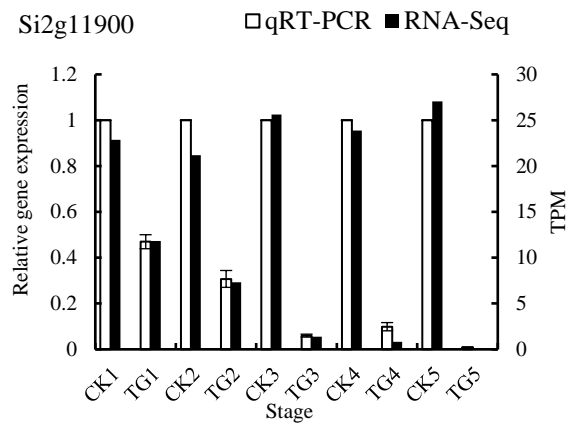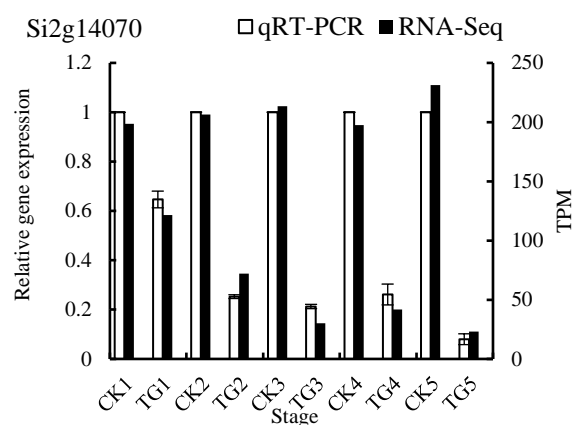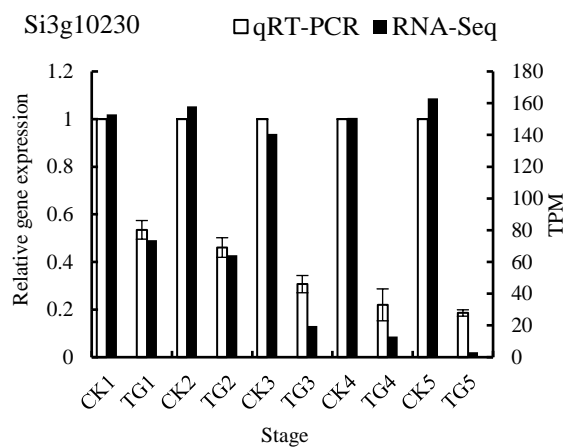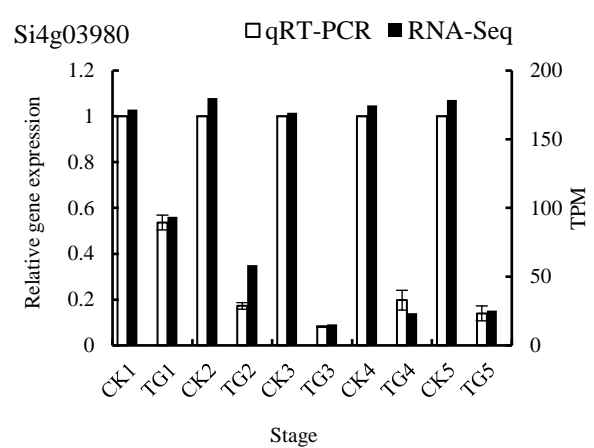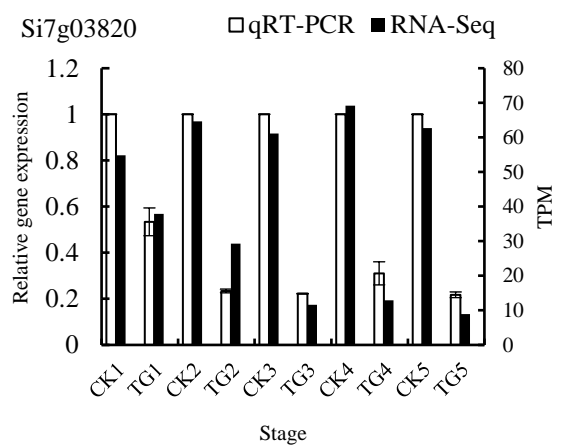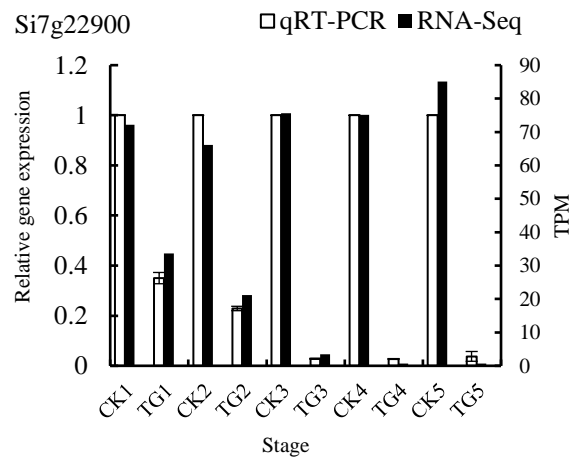

Supplementary Figure S3 Gene expression levels and qRT-PCR validation results

Supplement: Supplementary file 3 [file Image_3.pdf]
